# 60 years of *Cimicifuga racemosa* medicinal products: Clinical research milestones, current study findings and current development

**DOI:** 10.1007/s10354-016-0537-z

**Published:** 2017-02-02

**Authors:** Hans-Heinrich Henneicke-von Zepelin

**Affiliations:** Clinical Research, Schaper & Brümmer GmbH & Co. KG, Bahnhofstraße 35, 38259 Salzgitter, Germany

**Keywords:** Climacteric complaints, *Cimicifuga racemosa*, *Actaea racemosa*, Black Cohosh, Survival after breast cancer, Osteoporosis, Myoma, Cognition, Wechseljahresbeschwerden, *Cimicifuga racemosa*, *Actaea racemosa*, Traubensilberkerze, Überleben nach Brustkrebs, Osteoporose, Myom, Kognition

## Abstract

*Cimicifuga racemosa* (CR) extracts are important worldwide as therapy for menopausal symptoms. The first medicinal product from CR has been available since 1956 (Germany, Remifemin® [Schaper & Brümmer, Salzgitter, Germany], isopropanolic extract iCR). This review describes how CR developed, via clinical studies on safety (breast, breast cancer, endometrium, liver) and efficacy, into a successful and safe medicinal product in Germany, Europe and the world. In line with developing legal frameworks for medicinal products in Germany and Europe, clinical studies on CR were observational during the 50s and 70s, and controlled studies since the 80s. The first placebo-controlled study emerged 1986. From 2000 to 2015, a total of 28 clinical studies in Europe, America and Asia were published on the efficacy of CR. In these studies, 11,073 patients received a CR-based medicinal product, 93% thereof iCR. A meta-analysis of all nine placebo-controlled studies published until 2013 confirmed the reliable efficacy of CR-based medicinal products for menopausal symptoms.

## Zusammenfassung

Extrakte aus *Cimicifuga racemosa *(CR) haben zur Therapie von Wechseljahresbeschwerden weltweit an Bedeutung gewonnen. Das erste aus CR hergestellte Fertigarzneimittel steht seit 1956 zur Verfügung (Deutschland, Remifemin® [Schaper & Brümmer, Salzgitter, Deutschland], isopropanolischer Extrakt aus CR, iCR). Diese Übersichtsarbeit beschreibt, wie sich CR durch klinische Studien zur Sicherheit (Brust, Brustkrebs, Endometrium, Leber) und Wirksamkeit zu einem erfolgreichen und sicheren Arzneimittel in Deutschland, Europa und weltweit entwickelt hat. In Übereinstimmung mit der jeweiligen Entwicklung der gesetzlichen Rahmenbedingungen zu Arzneimitteln wurden klinische Studien zu CR in den 1950er- und 1970er- Jahren als beobachtende Studien durchgeführt. Seit den 1980er-Jahren folgten kontrollierte Studien, darunter die erste placebokontrollierte Studie 1986. Von 2000 bis 2015 wurden insgesamt 28 klinische Studien aus Europa, Amerika und Asien zur Wirksamkeit von CR veröffentlicht. In diesen Studien erhielten 11.073 Patientinnen ein CR-Arzneimittel, 93 % davon iCR. Eine Metaanalyse aller 9 bis 2013 veröffentlichten placebokontrollierten Studien bestätigte die zuverlässige Wirksamkeit von CR-Arzneimitteln gegen Wechseljahresbeschwerden.

## Short Overview

Approximately four out of five women suffer from menopausal symptoms (MPS) such as hot flushes and sweating, as well as from sleep disorders associated with these symptoms. Many women also suffer from psychological symptoms such as mood disorders, nervousness and irritability. For decades, extracts made from the rhizome of *Cimicifuga racemosa *(L.) Nutt. (CR, synonym for *Actaea racemosa *L., ‘Traubensilberkerze’, black cohosh) have been gaining worldwide importance as therapy for MPS. This herbal substance was originally used in North American folk medicine. The first medicinal product produced from black cohosh has been available since 1956 (Germany, Remifemin^®^ [Schaper & Brümmer, Salzgitter, Germany], isopropanolic extract iCR). At about the same time, Kupperman established his Menopause Index (KMI), which has since been used in numerous clinical studies with CR. From the 50s to the 70s, clinical research on CR followed the common practice of documenting and publishing clinical experiences. The German Medicines Act from 1976 enacted the obligation to provide proof of efficacy. Appropriately, controlled clinical trials followed in the 80s. From 1985 to 1987, the German Ministry of Health established standards on how to conduct clinical trials for drugs, i. e. medicinal products. Accordingly, the first randomized, placebo-controlled clinical trial with any CR extract was conducted (1986, iCR, KMI, Germany).

Good Clinical Practice (GCP) first came into effect in 1991 and was harmonized by the International Conference of Harmonization (ICH) in 1996. In 1994, Hauser published his Menopause Rating Scale (MRS) as a new tool to measure menopausal symptoms. A factor analysis established four MRS-I subscores in 2000; a self-assessment version appeared as MRS-II and is meanwhile available in 27 languages.

The first GCP compliant clinical study with CR in 1995 was dedicated to the question of dose–efficacy relationship (KMI, Poland). Daily doses of iCR extract from up to 127 mg CR proved to be safe. Without differentiating according to menopausal status, 40 mg already showed sufficient efficacy. However, perimenopausal women profited more from 127 mg than from 40 mg.

The second placebo-controlled clinical study for iCR followed from 2002 to 2005 (MRS, Germany). In addition to proof of efficacy, factors that influence efficacy were revealed and the first subtle hints of a supra-hypothalamic CNS (central nervous system) influence were found.

In 2003, the first placebo-controlled study with a *Cimicifuga* product from an ethanolic extract (Klimadynon^®^ [Bionorica, Neumarkt, Germany]) was published. The study strengthens the MRS by establishing the MRS-based effect size of hormone therapy (HT) for MPS compared to placebo (Czech Republic).

Controlled studies comparing iCR with other MPS therapies showed no differences in efficacy compared to hormone patches (2005, Italy) and tibolone, but iCR was superior regarding safety (2007, China). Another CR product (Remixin^®^ [Mikro-Gen, Istanbul, Turkey]) was compared to fluoxetine, with inferiority in the psychological component of the MPS and superiority in the KMI (2008, Turkey).

In 2005/2008, a clinical study clinically proved a CNS influence of iCR using positron emission tomography (USA). This is consistent with the pathophysiological tests in which CNS cortical activation accompanied menopausal hot flushes (2006, USA).

To date, the largest clinical study with CR (*n* = 6141, Germany) was published 2005/2007 and, for the first time, included over 12 months of safety data on patients (*n* = 736). iCR was compared with a combined product containing iCR and St. John’s wort (HP). iCR-HP showed an additional benefit on the psychological component of MPS. Proof of efficacy for this combination was also obtained using a placebo-controlled study (2006, MRS, Germany).

In 2006, a clinical study on safety at the endometrium with a 12-month CR therapy followed (Poland, Czech Republic).

The safety of iCR on breast tissue (mammography and fine-needle aspiration biopsy) was also substantiated with a clinical study: in 2007 alone, and in 2011 in a meta-analysis comparison with placebo, HT and tibolone (Sweden). Additionally, a pharmacoepidemiological cohort study demonstrated a 4.5-year longer recurrence-free survival after breast cancer for iCR users (2007, Germany).

In 2010, the Herbal Medicinal Product Committee (HMPC) of the European Medicinal Agency monograph attested the well-established use of drugs containing CR extract based on previously published studies on menopausal symptoms. According to the HMPC, there is no limit on the length of use, but after 6 months of therapy, a medical professional should be consulted. Breast cancer patients are not excluded from treatment of MPS with CR as long as a medical professional is consulted.

In 2011 the first meta-analysis was dedicated to the topic of liver safety. Of all five controlled clinical studies with iCR available at the time, the liver function test values were summarized where they had been raised. Evidence of liver toxicity was not found (international).

From 2000 to 2015, a total of 28 clinical studies in Europe, America and Asia were published on the efficacy of CR. In these studies, 11,073 patients received a CR drug, 93% thereof iCR. In 2013 a corrective reply to a Cochrane report on CR presented a complete meta-analysis of all nine placebo-controlled studies published until then; the report confirmed the reliable efficacy of CR drugs (international).

A recent study analysis evidenced that in women who were treated with iCR for MPS, myomas shrank in size compared to therapy with tibolone (2014, China). The latest placebo-controlled clinical study with iCR came from China. It showed improvement in sleep quality.

Future CR research may be dedicated to such topics as: mechanisms of action, possible extension of indications (e. g. prophylaxis for breast cancer recurrence) or additional uses (e. g. improvement of osteoporosis fractures or cognitive abilities). To date, the results of clinical research with CR confirm its safety and efficacy for menopausal symptoms and also provide valuable insights into additional uses, mechanisms of action and more.

## Kurzübersicht

Etwa 4 von 5 Frauen leiden während ihrer Wechseljahre an Beschwerden (WJB) wie Hitzewallungen, Schweißausbrüchen und damit assoziierten Schlafstörungen, etliche zusätzlich an psychischen Symptomen wie Verstimmungszuständen, Nervosität und Reizbarkeit. Seit Jahrzehnten gewinnt deren Therapie mit Extrakten aus dem Wurzelstock von *Cimicifuga racemosa *(L.) Nutt. (CR, synonym für *Actaea racemosa* L., Traubensilberkerze, Black Cohosh) weltweit an Bedeutung. Diese Arzneidroge stammt ursprünglich aus der Volksmedizin Nordamerikas. Das erste hieraus hergestellte Fertigarzneimittel steht seit 1956 zur Verfügung (Deutschland, Remifemin^®^ [Schaper & Brümmer, Salzgitter, Deutschland], isopropanolischer Extrakt, iCR). Etwa zeitgleich etablierte Kupperman seinen Menopause-Index (KMI, 1953–1959), der seither in diversen klinischen Studien zu CR eingesetzt wurde. In den 1950er- bis 1970er- Jahren folgte die klinische Forschung zu CR dem damals üblichen Vorgehen, dem Dokumentieren und Veröffentlichen von klinischen Erfahrungswerten. Das Arzneimittelgesetz 1976 führte die Pflicht zum Nachweis der Wirksamkeit ein, entsprechende kontrollierte klinische Studien folgten in den 1980er- Jahren. Das BMJFFG (Bundesministerium für Jugend, Familie, Frauen und Gesundheit) erließ 1985–1987 Grundsätze für die Durchführung der klinischen Prüfung von Arzneimitteln. Entsprechend wurde die erste randomiserte placebokontrollierte klinische Prüfung zu CR durchgeführt (1986, iCR, KMI, Deutschland).

Good Clinical Practice (GCP) trat erstmals 1991 und dann 1996 ICH-harmonisiert, also gemäß International Conference(s) of Harmonization, in Kraft. Hauser veröffentlichte 1994 seine Menopause Rating Scale MRS als neues Messinstrument von Wechseljahresbeschwerden, für die 2000 eine Faktorenanalyse die MRS-I-Subskalen etablierte und eine Selbstbeurteilungsvariante als MRS-II erschien, in mittlerweile 27 Sprachen verfügbar.

Die erste GCP-konforme klinische Studie zu CR widmete sich 1995 der Fragestellung zur Dosis-Wirksamkeits-Beziehung (KMI, Polen). Tagesdosen an iCR-Extrakt aus bis zu 127 mg CR erwiesen sich als sicher. Ohne Differenzierung nach Menopausestatus waren bereits 40 mg ausreichend wirksam. Doch perimenopausale Frauen profitierten von 127 mg mehr als von 40 mg.

In den Jahren 2002–2005 folgte die zweite placebokontrollierte klinische Prüfung zu iCR (MRS, Deutschland). Neben dem Wirksamkeitsbeleg arbeitete sie Einflussgrößen auf die Wirksamkeit heraus und ergab erste Hinweise auf einen suprahypothalamischen ZNS-Einfluss von iCR.

Die erste placebokontrollierte Studie zu einem Cimicifuga-Produkt mit einem ethanolischen Extrakt wurde 2003 publiziert (Klimadynon^®^ [Bionorica, Neumarkt, Deutschland]). Sie etablierte die MRS-basierte Effektgröße einer Hormontherapie der WJB im Vergleich zu Placebo und CR (Tschechien).

Kontrollierte Studien zum Vergleich von iCR mit anderen WJB-Therapien ergaben keinen Wirksamkeitsunterschied zu Hormonpflastern (2005, Italien) und zu Tibolon bei gleichzeitiger Überlegenheit in puncto Sicherheit (2007, erstmals China). Ein weiteres CR-Produkt (Remixin^®^ [Mikro-Gen, Istanbul, Türkei]) steuerte 2008 eine kontrollierte Studie im Vergleich zu Fluoxetin bei, mit Unterlegenheit in der psychischen Komponente der WJB und Überlegenheit im KMI (Türkei).

Eine klinische Studie belegte 2005/2008 den den ZNS-Einfluss von iCR klinisch per Positronenemissionstomographie (USA). Dies ist vereinbar mit pathophysiologischen Untersuchungen, in denen menopausale Hitzewallungen mit ZNS-kortikaler Aktivierung einhergingen (2006, USA).

Die bisher größte klinische Studie zu CR (*n* = 6141) stammt aus 2005 (Deutschland), davon 736 Patientinnen mit Sicherheitsdaten erstmals über 12 Monate. Sie verglich iCR mit einem Kombinationsprodukt aus iCR und Johanniskraut (HP) und zeigte für iCR-HP einen zusätzlichen Benefit-Effekt auf die psychische Komponente der WJB. Auch für diese Kombination erfolgte der Wirksamkeitsbeleg mittels placebokontrollierter Studie (2006, MRS, Deutschland).

Im Jahr 2006 folgte eine klinische Studie zur Sicherheit am Endometrium unter 12-monatiger CR-Therapie (Polen, Tschechien).

Die iCR-Sicherheit am Brustgewebe (Mammographie und Feinnadelbiopsie) wurde auch mittels klinischer Studie untermauert: 2007 allein und 2011 in metaanalytischem Vergleich zu Placebo und Tibolon (Schweden). Zudem ergab eine pharmakoepidemiologische Kohortenstudie ein 4,5 Jahre längeres rezidivfreies Überleben für iCR-Anwenderinnen nach Brustkrebs (2007, Deutschland).

2010 attestierte das HMPC (Herbal Medicinal Product Committee der European Medicinal Agency) per Monographie einen Well Established Use von CR-Extrakt-haltigen Arzneimitteln auf Basis der bis dahin publizierten Studien bei WJB. Gemäß HMPC beinhaltet die Anwendungsdauer keine zeitliche Begrenzung, nach 6 Monaten Therapiedauer soll jedoch ärztlicher Rat eingeholt werden. Die Behandlung mit CR von WJB schließt auch Brustkrebspatientinnen nicht aus, sofern ein entsprechender Rat vom Arzt eingeholt wird.

Die erste Metaanalyse widmete sich 2011 dem Thema Lebersicherheit. Sie fasste die Leberfunktionswerte aller 5 bis dahin verfügbaren kontrollierten klinischen Studien zu iCR zusammen, in denen diese erhoben worden waren. Hinweise auf eine Lebertoxizität ergaben sich nicht (international).

Von 2000 bis 2015 wurden insgesamt 28 klinische Studien aus Europa, Amerika und Asien zur Wirksamkeit von CR veröffentlicht. In diesen Studien erhielten 11.073 Patientinnen ein CR-Arzneimittel, 93% davon iCR. Im Jahr 2013 erschien in Korrektur eines Cochrane Berichts eine vollständige Metaanalyse aller 9 bis dahin veröffentlichten placebokontrollierten Studien und bestätigte eine zuverlässige Wirksamkeit von CR-Arzneimitteln (international) gegen WJB.

Eine aktuelle Studienauswertung ergab, dass sich Myome bei den Frauen verkleinerten, die ihre WJB mit iCR im Gegensatz zu Tibolon behandelten (2014, China). Die jüngste placebokontrollierte klinische Studie zu iCR stammt aus China. Sie zeigte eine Verbesserung der Schlafqualität.

Zukünftige CR-Forschung dürfte sich den Themen Wirkungsmechanismen, möglichen Indikationserweiterungen (z.B. Rezidivprophylaxe bei Brustkrebs) oder Zusatznutzen (z.B. Verbesserung der Frakturraten bei Osteoporose oder kognitiven Fähigkeiten) widmen. Die bisherigen Fakten aus der klinischen Forschung (KliFo) zu CR bestätigen die Sicherheit und Wirksamkeit bei WJB, liefern aber auch wertvolle Ansatzpunkte hinsichtlich Zusatznutzen, Wirkmechanismen und mehr.

## Introduction

The current issue of the *Wiener Medizinische Wochenschrift* is dedicated to therapy with products containing herbal ingredients. Rigorously and rationally investigated herbal medicinal products are available for use in humans. Examples thereof are products containing herbal medicinal preparations (extracts) made from the rhizome of *Cimicifuga racemosa* (L.) Nutt. (CR, synonym for the botanical reclassification name *Actaea racemosa *L.), Traubensilberkerze, Black cohosh). 60 years ago, in 1956, the career of the first controlled regular medicinal product using this raw material commenced after centuries of its use in North American folk medicine. During these 60 years, extracts made from CR have gained worldwide importance as therapy for menopausal symptoms. In consideration of this anniversary, the current article describes the progress of clinical research evidence on CR during the decades since its official birthdate (Table 1).

**Table 1:** Curriculum vitae. Milestones of clinical research in the career of the first industrial herbal medicinal product containing an extract of *Cimicifuga racemosa* (CR)

Year	Milestone	Country/region
**1956**	Year of birth of Remifemin®	Germany
1956 to 1970s	Documentation of clinical experiences	Germany
1980s	First randomized controlled clinical trials according to 1976 Medicines Act	Germany
**1986**	First placebo-controlled clinical trial on iCR	Germany
1989	German Commission E monograph on CR	Germany
1991	Good Clinical Practice	Europe
1995	First clinical dose–efficacy study	Poland
2003	First placebo-controlled study on another CR extract; MRS-based effect size of hormone therapy (HT)	Czech Republic
2002 to 2005	Second placebo-controlled study on iCR confirms efficacy, identifies confounders influencing the effect size, first subtle hints of supra-hypothalamic CNS influence	Germany
2005	Controlled study in comparison to hormone patches	Italy
2005/2008	Clinical evidence on CNS influence of iCR by PET	USA
2007	Controlled study in comparison to tibolone, first study in China	China
2007	Clinical study on iCR safety in breast tissue	Sweden
2007	Pharmacoepidemiological cohort study on recurrence-free survival after breast cancer	Germany
2010	Monograph of the Herbal Medicinal Product Committee at the European Medicines Agency concludes well-established use of CR-based medicines for alleviating menopausal symptoms, does not limit the duration of use, allows the use in breast cancer survivors if medically advised	Europe
2011	Meta-/reanalytic safety comparison of iCR with placebo, tibolone and HT regarding breast density	Sweden
2011	Meta-analysis on liver safety	Europe, China
2005 to 2015	More clinical studies on iCR in Europe, America, Asia	International
2012	Cochrane Report on black cohosh (*Cimicifuga ssp.*) for menopausal symptoms	Australia
2013	Corrective reply to Cochrane Report, complete meta-analysis of all nine placebo-controlled studies on efficacy	International
2013	Systematic review on efficacy (18 studies) and safety (35 studies); conclusive evidence on efficacy if of licensed product quality	Germany
2014	Supplementary benefit in myoma patients	China
2015	Most recent placebo-controlled study; improvement of sleep quality	China
**2016**	Anniversary. 11,073 patients in 28 clinical studies on efficacy since 2000 (10,883 investigated with a medicinal product of licensed quality, 89% thereof iCR)	Total

*iCR* isopropanolic CR extract, *MRS* Menopause Rating Scale, *CNS *Central nervous system, *PET* Positron emission tomography, *HT* Hormone therapy

## Menopausal symptoms

More than 1.2 billion women in the world will go through menopause by the year 2030 [1]. For a few women, this natural process of aging runs its course asymptomatically, while approximately four out of five women suffer from MPS; this includes MPS caused by surgery, chemotherapy or pelvic radiation. Worldwide, between 50 and 85% of these women experience severe and disabling symptoms, which significantly affect their wellbeing and quality of life [1–3]. MPS comprise hot flushes and (night) sweats, as well as sleep disturbances associated with these symptoms. Many women also suffer from psychological symptoms such as mood disorders, nervousness and irritability. The duration of these bothersome menopausal symptoms is estimated to be about 8 to 12 years [4, 5]. These are important reasons for seeking medical attention and for receiving therapy.

## Product birthdate 1956

The first medicinal product produced from black cohosh has been available since 1956 (Remifemin®). Two galenic formulations for oral intake were launched: tablets containing the isopropanolic extract iCR and a solution containing an ethanolic extract. Shortly thereafter, the first publication described clinical experiences in treating climacteric complaints with the new product [6]. At about the same time, Kupperman established his Menopause Index (KMI, 1953–1959) [7, 8]. It is calculated as the sum of severity coefficients of the 11 most common symptoms with assigned symptom-specific weight factors (Table 2). The KMI has since been used in numerous clinical studies, also with iCR.

**Table 2:** Kupperman Menopause Index (KMI). Original wording, coding and example [7, 8]

Symptoms	Factor	Severity	Numerical Conversion
Vasomotor	4	+ = 3	12
Paraesthesia	2	M = 2	4
Insomnia	2	M = 2	4
Nervousness	2	S = 1	2
Melancholia	1	0 = 0	0
Vertigo	1	0 = 0	0
Weakness (fatigue)	1	M = 2	2
Arthralgia and myalgia	1	S = 1	1
Headaches	1	+ = 3	3
Palpitation	1	M = 2	2
Formication	1	S = 1	1
Menopausal index			31
Code			
0 – none = 0 S – slight = 1 M – moderate = 2 + – marked = 3			

## Clinical studies in the 50s to the 70s

From the 50s to the 70s, clinical research on CR followed the common practice of documenting and publishing clinical findings. Practicing gynaecologists and general practitioners, as well as outpatient departments of hospitals documented their experiences per patient on simple case record forms reflecting medical routine. Thirteen publications with a total of 1810 patients resulted from these systematic clinical observations (Table 3). However, their relevance for the total body of evidence on CR has decreased due to the availability of new evidence complying with modern standards for clinical studies.

**Table 3:** Observational clinical studies on *Cimicifuga racemosa* during the first decade after launch of Remifemin®. (A reference list of these old studies is available from the author on request)

Author, year of publication	No. of patients
Kesselkaul, 1957	62
Krämer and Geisenhofer, 1958	252
Schotten, 1958	22
Földes, 1959	82
Stefan, 1959	94
Stiehler, 1959	53
Brücker, 1960	517
Görlich, 1960	88
Heizer, 1960	110
Starfinger, 1960	105
Görlich, 1962	258
Langfritz, 1962	73
Schildge, 1964	135
Total	1810

## Changes in the 80s towards controlled studies

Whereas the German Medicines Act from 1961 obliged manufacturers to notify any medicinal product to a list only, the thoroughly revised German Medicines Act from 1976 enacted the manufacturer’s obligation to provide proof of quality, safety and efficacy of existing and new medicinal products. A transition period was granted for existing drugs until 1990. In the first years after this new legislation, in-depth discussions were held to figure out how to prove these three aspects. Besides the scientific necessities, this debate also included emotional aspects such as the accusing headline on the cover page of the journal *Der Spiegel* in 1978, stating “Menschenversuche in deutschen Krankenhäusern” (Experiments on Humans in German Hospitals; http://www.spiegel.de/spiegel/print/d-21112940.html). Scientific societies discussed possible clinical study designs from methodological perspectives, e. g. the German Association of Medical Documentation, Informatics and Statistics (GMDS), who dedicated their annual congress to the motto “Therapy Studies—Planning, Realization, Results, Impact” in 1981. Medical scientists and biometricians stated to appreciate randomized controlled trials (RCTs) which appropriately followed in the 1980s for numerous medicinal products, including head-to-head-studies of iCR against reference therapies [9, 10]. When answers to the methodological question “how” seemed to have become settled, first detailed rules enacted a regulatory framework for clinical trials, i. e. from 1985 to 1987, the German Ministry of Health established standards on how to conduct clinical trials for drugs, i. e. medicinal products [11]. Accordingly, the first double-blind, randomized, placebo-controlled clinical trial with CR was also conducted during these years (report 1986, publication 1987, Germany) [12]. This study investigated iCR for its efficacy as measured by the KMI in 80 patients. It revealed a significant superiority of iCR to placebo for alleviating climacteric complaints in the 12-week treatment period, with an onset of efficacy before the first post-baseline visit at 4 weeks [12].

Consequently, the German Commission E approved a positive benefit–risk balance of *Cimicifuga racemosa* for the treatment of climacteric neurovegetative complaints [13].

## GCP and the MRS enter the stage in the 90s

Good Clinical Practice (GCP) first came into effect in Europe in 1991 and was harmonized by the International Conference(s) of Harmonization (ICH) in 1996 (http://www.ich.org/fileadmin/Public_Web_Site/ICH_Products/Guidelines/Efficacy/E6/E6_R1_Guideline.pdf). GCP is an international ethical and scientific quality standard for designing, conducting, recording and reporting trials that involve the participation of human subjects. Compliance with this standard provides public assurance that the rights, safety and wellbeing of trial subjects are protected and are consistent with the principles that have their origin in the Declaration of Helsinki, and that the clinical trial data are credible. Although it started as a guideline, it was subsequently implemented to mandatory law by GCP directives of the EU council (2001/20/EC) and the EU commission (2005/28/EC).

In 1994, Hauser published his Menopause Rating Scale MRS as a new tool to measure menopausal symptoms [14]. This was a reaction to criticism of the KMI regarding its weighing of symptoms and the incompleteness of its profile of menopausal symptoms. The MRS used a vocabulary of modern language to describe the symptoms assessed by the ten items of the score (Table 4). It took another 6 years to make this new scale ripen. In particular, an additional advantage arose with the establishment of the four MRS-I subscores by factor analysis in a large-scale clinical study in several thousand patients in 2000 ([15]; Table 4). Additionally, a self-assessment version appeared as MRS-II [16] and is meanwhile available in 27 languages.

**Table 4:** Menopause Rating Scale (MRS-I). The investigator assesses each of ten items on a scale ranging from 0 (no complaints) to 1 (severe symptoms) in increments of 0.1. Subsequently to the original scale [14], subscores were established by factor analyses [15]. Total score and subscores are calculated as the mean of the comprising items

Item	Pertaining to subscore	Symptom group	Climacteric symptoms
1	HOT FLUSHES	Hot flushes, sweating	Sensation of rising heat, outbreaks of sweating (frequency/intensity per 24 h)
2	SOMA	Cardiac symptoms	Palpitations, racing heartbeat, irregular beats, tightness in chest
3	HOT FLUSHES	Sleep disorders	Difficulty in falling asleep, difficulty in remaining asleep through the night, waking too early
4	PSYCHE	Depressive moods	Despondency, sadness, tearfulness, lack of drive, mood fluctuations
5	PSYCHE	Nervousness, irritability	Nervousness, inner tension, aggressivity
6	PSYCHE	Impaired performance/memory	Susceptibility to physical and mental exhaustion, poor concentration, forgetfulness
7	ATROPHY	Disorders of sexuality	Reduced libido, sexual activity and satisfaction
8	ATROPHY	Urinary symptoms	Symptoms during urination, frequent need to pass urine, accidential incontinence
9	ATROPHY	Vaginal dryness	Feeling of dryness of the vagina, symptoms during sexual intercourse
10	SOMA	Joint and muscle symptoms	Pain predominantly affecting the finger joints, rheumatic symptoms, itching

The first GCP compliant clinical study with black cohosh in 1995 investigated iCR in a multicentre setting in Poland and was dedicated to the question of the dose–efficacy relationship. 152 patients suffering from menopausal complaints were randomly assigned to the treatment groups and assessed using the KMI and routine safety parameters including laboratory parameters. Daily doses of iCR extract of up to 127 mg CR proved to be safe and did not influence oestrogenic parameters such as luteinizing hormone (LH), follicle stimulating hormone (FSH), serum oestradiol (E2), sex hormone-binding globulin (SHBG), prolactin or vaginal cytology [17]. Without differentiating according to menopausal status, iCR extract from 40 mg CR already showed sufficient efficacy in terms of significant improvement of the KMI in comparison to baseline [17]. The response rates (KMI < 15) were 72 and 90% after 3 and 6 months of treatment, respectively. However, perimenopausal women profited more from iCR extract from 127 mg CR than from 40 mgCR [18].

## Several placebo-controlled or reference-controlled studies in the 2000s

The second double-blind randomized placebo-controlled clinical study for iCR followed from 2002 to 2005, in a multicentre setting in Germany. 304 patients suffering from menopausal complaints participated and were assessed using the MRS, its MRS subscores and routine safety parameters [19]. In addition proving efficacy again, this study revealed factors that influence efficacy and found first subtle hints of a supra-hypothalamic CNS influence of iCR [19]. Women during their first years of menopausal complaints showed a better superiority to placebo in terms of improvement of their symptoms than those who had been suffering from their complaints for several years [19]. The effect size was 0.03 to 0.05 MRS units, which is similar to HT study results (0.036 MRS units, see below), thus showing its clinical relevance. Besides superiority in the MRS total score as the primary efficacy endpoint, the MRS subscore “HOT FLUSHES” (also named vasomotor symptoms, VMS) was the most prominent set of symptoms sensitive to improvement by iCR. This “key competence” of iCR comprises the MRS description “hot flushes, sweatings and associated sleep disorders” (Table 4). Adverse events and other safety parameters were similar in both treatment groups [19].

At approximately the same time, the first placebo-controlled study with a *Cimicifuga* product from an ethanolic extract (Klimadynon®) was performed in the Czech Republic and published in 2003. Only 62 patients were evaluable for efficacy after having been randomly allocated to receive a placebo, the CR extract or hormone therapy. Unfortunately for the investigators, their CR effect (0.037 MRS units) narrowly missed the significance level due to this too small sample size. However, the particular contribution of this study is that it strengthens the MRS by establishing the MRS-based effect size of hormone therapy (HT) for menopausal symptoms, i. e. 0.036 MRS units compared to placebo [20]. Additionally, CR and HT led to beneficial effects on serum parameters of bone metabolism and an increase in vaginal superficial cells, but, in contrast to HT, there was no CR effect on endometrial thickness.

Subsequent controlled studies published in 2005 and 2007 that compared iCR (Remifemin®) with other MPS therapies showed no relevant differences in efficacy compared to oestradiol patches [21] and tibolone, but iCR was superior regarding safety [22]. In detail, the comparison to oestradiol patches [21] for a 3-month treatment was performed in 64 patients in Italy. The efficacy parameters (hot flush diary, Greene scale) significantly improved over time, without relevant differences between the treatment groups. Serum hormone levels (LH, FSH, oestradiol, prolactin), safety-relevant serum enzymes for liver function and endometrial thickness did not change over time in the iCR-group. Interestingly in contrast, LDL levels (low density lipoprotein) decreased and HDL levels (high density lipoprotein) increased over time in the iCR-group [21]. The comparison of iCR to tibolone [22] was a double-blind, randomized controlled study and the first study on a CR-based herbal medicinal product with licensed pharmaceutical quality in an Asian population, particularly in China. This study was necessary and pivotal for the regulatory approval of iCR in that country. As yet, no other CR extract has been approved by the Chinese regulatory authority (SFDA). 244 patients were included and applied either iCR or tibolone for 3 months. The well-established Chinese version of the KMI served as the efficacy parameter and significantly improved over time, without relevant differences between the treatment groups, even for moderate to severe symptoms. The KMI responder rate was also similar in both groups (84% and 85%). The safety evaluation showed a good safety and tolerability profile for both groups; however, there was a significantly lower incidence of adverse events (*p* < 0.0001) in favour of the herbal treatment. In particular, none of the postmenopausal iCR patients experienced vaginal bleeding in contrast to tibolone (17 cases). As a result, the primary endpoint (benefit–risk balance), which had been prespecified in the study protocol as the combination of the Mann–Whitney values of the KMI and the frequency of adverse events, also showed superiority of iCR to tibolone (*p* = 0.009).

In 2008, another CR product (Remixin®) contributed a controlled study comparing CR to fluoxetine, with inferiority in the psychological component of the MPS and superiority in the KMI (Turkey) [23].

## It’s in the brain: pathophysiology of hot flushes/sweating and iCR effects seem to match

Pharmaceutical products with proven efficacy, safety and reproducible pharmaceutical quality are a matter of interest regarding their mode of action. Hence, iCR was also investigated for pharmacodynamic effects. Several preclinical studies argue for an oligo-factorial mode of action of CR extracts (see below), including effects on CNS-relevant systems. Clinically, a milestone was reached in 2005 by a poster at the annual conference of the North American Menopause Society and 2008 by the corresponding full publication, i. e. a clinical study proved a CNS influence of iCR using positron-emission tomography (USA) [24]. The authors had treated postmenopausal women with iCR for 3 months and found changes in µ‑opioid receptor availability in distinct brain regions. In detail, iCR increased opioid-binding in regions known to be relevant for emotional and cognitive functions (thalamus, nucleus accumbens, posterior and anterior cingulum) and decreased the opioid binding in the dorsoanterior cingulum and the anterior insular cortex. Although these regions are known to be oestrogen-sensitive, the iCR effects were not oestrogenic because iCR alone did not change the frequency of the LH pulse, whereas iCR in combination with the opioid antagonist naloxone did decrease this frequency [24].

Interestingly and consistently, some of these regions were identified during those years as also being in the pathophysiology of hot flushes and sweating [25]. In detail, Freedman et al. compared brain activation in symptomatic postmenopausal women and asymptomatic eumenorrheic women in their controlled laboratory study using magnetic resonance imaging. Areas of activation during hot flashes in symptomatic women included the insula and anterior cingulate cortex. Sweating in eumenorrheic women was associated with activity in the anterior cingulate and superior frontal gyrus. The authors concluded that thermoregulation in humans appears to be represented in a distributed cortico–subcortical network rather than in a single localized structure [25].

This matching of Reame’s [24] and Freedman’s data [25] accords with preclinical results. CR/iCR contains substances which bind to serotonin, dopamine, GABA and µ‑opioid brain receptors leading to receptor-mediated functional activity [26–30]. CR/iCR modulates the ratio of cerebral monoamines and metabolites [31] as well as brain activity (EEG) [32], i. e. serotonergic and dopaminergic systems and µ‑opioid receptor availability [29]. iCR ameliorates an ovariectomy-induced decrease in a serotonin dorsal raphe–preoptic hypothalamus pathway [33]. iCR recovered the activity of neurons in the preoptic area in the hypothalamus which were impaired by oestrogen deprivation [34, 35]. After iCR treatment, neurons recovered their sensitivity to temperature changes and were identified as active neurons [34]. These data suggest that alleviation of VMS by CR/iCR is not caused by oestrogen-agonistic effects. The hypothesis of an association between CNS receptor-mediated effects and an efficacious relief of VMS has become more plausible, since iCR binds to CNS receptors and modulates brain function and metabolism involved in thermoregulation. Future research is needed to verify this hypothesis.

## Upscale of sample size and duration of clinical studies

The largest clinical study with CR to date (*N* = 6141 female patients) was presented at the annual conference of the German Menopause Association in 2005 and fully published in 2007. For the first time, it included over 12 months of safety data on patients (*N* = 736) [36]. 1287 gynaecologists [36] in Germany included women suffering from and treated for their VMS or climacteric mood symptoms or both into this prospective, controlled, open-label, non-interventional study. iCR was compared with the combined product containing iCR and St. John’s wort (HP). Patients were followed up for 6 months, optionally 12 months if treated for this duration. The primary effectiveness variable was the MRS subscore “psyche” at month 3 and evaluated by ANCOVA (analysis of covariance). This MRS subscore comprises climacteric depressive moods, nervousness, irritability and impaired cognitive functions (Table 4). The total MRS score improved with both regimens during the first 3 months, further improved over time during the subsequent 3 months, and did not change anymore during months 6 to 12. iCR-HP was superior to iCR in the primary endpoint, i. e. showed an additional benefit in the psychological component of MPS, i. e. climacteric mood complaints [36]. The study did not find any suspicion of liver-related side effects. The rate of possibly treatment-related adverse events was 0.16%, all non-serious. The global assessment of tolerability revealed this to be good or very good in more than 90% of the patients [36].

Proof of efficacy for this combination was also achieved using a double-blind, randomized, placebo-controlled study (2006) [37]. The study included 301 patients for treatment with the iCR-HP combination for 4 months in Germany and found a 50% MRS improvement by this herbal treatment in comparison to 20% in the placebo group (*p* < 0.001). The study additionally assessed the climacteric mood complaints using the Hamilton Depression Scale (HAMD) and revealed a 42% HAMD improvement with the iCR-HP medication in comparison to 13% in the placebo-group (*p* < 0.001). The frequency and pattern of adverse events, safety laboratory results and the clinical global impression of tolerability were similar in both treatment groups [37].

## Clinical safety at endometrium and breast

In 2006, a clinical study on safety at the endometrium with a 12-month CR therapy followed. This prospective, open-label, multicentre study in Poland and the Czech Republic included 400 patients, assessed them by ultrasonography and did not find any case of endometrial hyperplasia or any increase of the mean of the endometrial thickness over time [38].

The safety of iCR on breast tissue was also substantiated with a clinical study: in 2007 alone (*N* = 64) [39] and in 2011 in a meta-analysis together with a preceding study comparing placebo (*N* = 53), HT (*N* = 43) and tibolone (*N* = 49) [40]. Both studies were performed in the same setting at the Karolinska Hospital in Stockholm, Sweden. Blinded observers compared the baseline status of mammography (breast density) and fine-needle aspiration biopsy (breast cell proliferation assessed as the frequency of Ki-67-positive cells) with the status after 6 months of treatment. Breast density remained unchanged in the placebo group and the iCR group (0% and no case more than 5%) but significantly increased (*p* < 0.001) in the HT group (+14.3% and 27 cases more than 5%) and the tibolone group (+2.3% and 10 cases more than 5%) [40]. Furthermore, there was no increase in breast cell proliferation upon iCR treatment, i. e. no increase in the mean/median proportion of Ki-67-positive cells [39]. The mean/median change in ultrasonographically investigated endometrial thickness was 0 [39]. These clinical study data on iCR do not give any indication of adverse effects on healthy breast tissue or any endometrial safety concerns [39].

In 2007, a pharmacoepidemiological cohort study demonstrated a 4.5 year longer recurrence-free survival after breast cancer for iCR users [41]. This study examined the data of breast cancer patients treated at general, gynaecological and internal facilities linked to a medical database in Germany. The impact of treatment with iCR following diagnosis was analysed by Cox proportional hazards models, controlling for age and other confounders. Of 18,861 patients, a total of 1102 had received an iCR therapy (either iCR alone or the iCR-HP combination). The mean overall observation time was 3.6 years. iCR exposure was not associated with an increase in the risk of recurrence but was associated with prolonged disease-free survival. 2 years after initial diagnosis, 14% of the control group had developed a recurrence, while the iCR group reached this proportion after 6.5 years. The primary Cox regression model controlling for age, tamoxifen use and other confounders demonstrated a protractive effect of iCR on the rate of recurrence (hazard ratio 0.83, *p* = 0.039). This effect remained consistent throughout all variations of the statistical model, including subgroup analyses. TNM status was unknown but did not bias the iCR treatment decision (due to climacteric complaints) as investigated separately. Once in a while, this study is criticized for this TNM uncertainty in the dataset. However, due to the independency of the treatment decision from the TNM status, the missing of TNM information is not a source of bias for the comparison of iCR-users with nonusers regarding the endpoint of this study. Notably, the missing TNM data even impaired the power of the study to detect any group difference on a significant level because its impact cannot be deducted from the overall variance of the endpoint. But if a detected difference is already significant, such impairment of power becomes irrelevant. In contrast, if endpoint and reason for treatment allocation depend on the same confounder, this can cause bias and should not suffer from missing data and should be included into the regression model (cofactor/covariate or stratification). This applies for the confounders age and tamoxifen use, and the study’s analyses appropriately cared for this [41]. In particular, the beneficial recurrence protractive effect of iCR occurred in tamoxifen-users and also in tamoxifen-nonusers [41].

## HMPC monograph and meta-analyses

In 2010, the HMPC attested the well-established use of medicinal products containing CR extract based on previously published studies on menopausal symptoms [42]. An essential precondition of regulatory authorities’ approval of products based on such monographs is the proof and approval of its product-specific pharmaceutical quality. The pharmaceutical characteristics of HMPC-accepted herbal medicinal products made from *Cimicifuga racemosa* comprise the “quantitative and qualitative composition” requiring “dry extract (drug–extract ratio, DER 5‑10:1), extraction solvent ethanol 58%” or “dry extract (DER 4.5-8.5:1), extraction solvent ethanol 60%” or “dry extract (DER 6‑11:1), drug extraction solvent isopropanol 40%”. Notably, the HMPC monograph does not limit the length of the use of CR extracts, but after 6 months of therapy, a medical professional should be consulted. The reason behind this is that women who usually suffer from MPS are in a certain age range when they should see their doctor anyway, in order to avoid overseeing any serious disease. Also, breast cancer patients are not excluded from the treatment of MPS with CR as long as a medical professional is consulted. This is actually a matter of course, because physicians who treat cancer patients should know every medicinal product their patients take [42].

The clinical data on the safety of CR extracts in endometrium, liver, breast and breast cancer were further augmented by numerous supportive preclinical studies in animals and cell cultures. For preclinical details, refer to the assessment report of the HMPC monograph on CR [42].

In 2011 the first meta-analysis was dedicated to the topic of liver safety [43]. In all five controlled clinical studies with iCR available from any country at that time, the liver function test values were summarized where they had been raised. The dataset comprised 1117 peri- or postmenopausal women who treated their symptoms with iCR for 3 to 6 months. The dosages of the extracts corresponded to 40–128 mg herbal substance per day. The meta-analysis did not find any group difference regarding the safety-relevant serum enzymes for liver function, particularly also neither at the highest dosage nor upon the longest duration of exposure. Evidence of liver toxicity was not found [43]. This accords with the safety results of a recent systematic review [44] and its update [45], which did not find any case of hepatotoxicity among the more than 12,000 patients in all clinical studies on the safety of CR-based study medication.

**Fig. 1:**
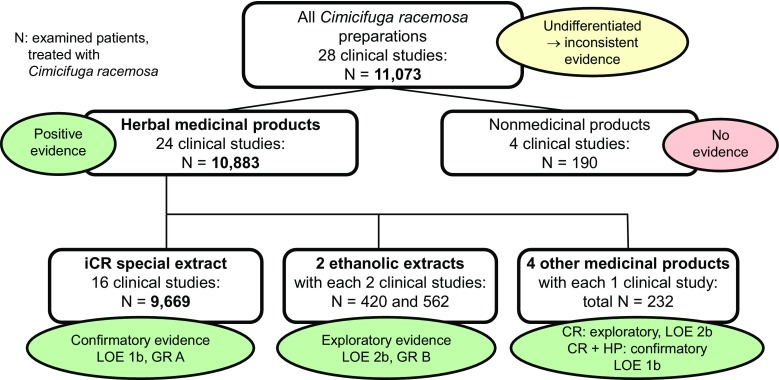
Efficacy data sets from clinical studies on *Cimicifuga racemosa* (*CR*) from 2000 to 2015. *iCR* isopropanolic extract of CR rootstock, *HP* *Hypericum perforatum, LOE* Oxford Level of Evidence, *GR* Oxford Grade of Recommendation

This systematic review [44] and its update [45] certified an Oxford level of evidence 1a (safety) or 1b (efficacy) and grade of recommendation A for the iCR, with lower level of evidence and grade of recommendation for other CR extracts. Particularly the efficacy aspect of this systematic review deserves further attention, because the reviewer figured out a very important key point for interpretation of efficacy results of all recent studies: conclusive evidence on efficacy if of licensed-product quality, inconclusiveness only if other quality was investigated. From 2000 to 2015, a total of 29 clinical studies in Europe, America and Asia were published on the efficacy of CR. In these studies 10,049 patients received a CR-based medicinal product, 93% thereof iCR (Fig. 1; [44–46]).

In 2013 a corrective reply to the Cochrane Report on black cohosh—*Cimicifuga ssp*. in 2012 presented a complete meta-analysis of all nine placebo-controlled studies published till then; the report confirmed the reliable efficacy of CR-based medicinal products. The result of this meta-analysis estimates the standardized effect size in comparison to placebo to be 0.385 standard deviations (*p* < 0.0001; Table 5; [47]).

**Table 5:** Placebo-controlled studies on *Cimicifuga racemosa* for alleviating natural menopausal complaints. For bibliographic details of these studies see Beer et al. [47]

Author, year	Licensed medicinal product	Number of patients	Standardized difference in means	95% confidence interval
Frei Kleiner 2005	Yes	122	−0.090	−0.461 to 0.281
Geller 2009	No	43	0.170	−0.408 to 0.748
Amsterdam 2009	No	28	0.430	−0.286 to 1.146
Newton 2006	No	164	−0.270	−0.578 to 0.038
Osmers 2005	Yes	286	−0.394	−0.626 to −0.162
Stoll 1987	Yes	50	−1.020	−1.586 to −0.454
Wuttke 2003	Yes	40	−0.639	−1.260 to −0.018
Kaiser 2008	Yes	120	−0.617	−0.975 to −0.259
Li Yilin	Yes	77	−0.792	−1.246 to −0.338
**Meta-analysis summary of efficacy**	**Any**	**930**	**−0.385**	**−0.514 to −0.255** **(p < 0.0001)**

## Supplemental benefits and upfront potential

A recent study analysis evidenced that in women who were treated with iCR for MPS, the myomas shrank in size compared to therapy with tibolone (2014) [48]. The dataset of this supplemental analysis was a subset of the randomized controlled study comparing iCR and tibolone in menopausal complaints in China [22] and comprised all 62 patients with at least one uterine fibroid at onset of treatment. The size of the fibroids had been measured by transvaginal ultrasonography. The median myoma volume decreased with iCR for 3 months by as much as −30% (*p* = 0.016), but increased with tibolone by +4.7%. The latter corresponds to the +4.4% that can be expected for untreated myoma patients of similar age.

Data from four clinical trials [49–52] hint at beneficial effects of CR on bone metabolism and bone mineral density which might help to reduce the cumulative dose of HT for prophylaxis of osteoporosis.

The latest placebo-controlled clinical study with iCR came from China. It showed the improvement of sleep quality in postmenopausal women with sleep disturbances [53].

## Adverse drug reactions

The profile of adverse drug reactions (ADR) which emerged from all the clinical studies and from pharmacovigilance monitoring is summarized in the package insert leaflet and in the summary of product characteristics of licensed medicinal products according to the HMPC monograph and comprises for iCR: Rarely: gastrointestinal symptoms, allergic skin reactions, facial or peripheral oedema, increase of liver enzymes in the serum, increase of body weight. Very rarely, cases of liver toxicity were reported during the use of *Cimicifuga *products. However, a causal relationship has not yet been confirmed.

To date, none of all the clinical studies on the safety of CR-based study medication found any case of hepatotoxicity among their more than 12,000 participating patients.

## Long-term treatment

The duration of use of medicinal products intended for non-life-threatening diseases is directly linked to a positive risk–benefit assessment based on clinical data over distinct exposure periods according to the ICH-E1 guideline (http://www.ema.europa.eu/docs/en_GB/document_library/Scientific_guideline/2009/09/WC500002747.pdf). For a long-term treatment, this guideline requires that the number of patients monitored for ADR in clinical studies:

- should be at least 300–600 treated for 6 months. This principle is fulfilled already by the 6141 patients exposed to iCR for at least 6 months in the study by Briese et al. [36] and several additional hundreds of patients in all the other clinical studies on iCR (for a complete list see [44] and its update [45]).
- should be at least 100 treated for 12 months. This principle is fulfilled by the 736 patients exposed to iCR for 12 months in the study by Briese et al. [36].
- should be at least 1500 treated for any duration. This principle is fulfilled by the total number of about 12,000 patients exposed to iCR in interventional and non-interventional clinical studies [36, 44, 45].

One may discuss whether the chapter “exceptions” in ICH-E1 applies, e. g. if concerns had arisen from animal studies. However, the chronic toxicity studies available for the iCR products did not reveal such concern (unpublished preclinical study reports available at the regulatory authorities who granted marketing authorizations of these products).

Sufficient clinical study data are available, at least for iCR among the CR extracts, to fulfil the ICH-E1 set of principles for a safe long-term treatment (chronic or repeated intermitted use longer than 6 months).

Independently from the sufficient extent of preclinical and clinical data supporting the safe use of black cohosh, all menopausal women should seek routine medical advice every 6 to 12 months, to avoid other relevant diseases being overlooked.

## Future

Future CR research may be dedicated to such topics as: mechanisms of action, possible extension of indications (e. g. prophylaxis for breast cancer recurrence) or additional uses (e. g. improvement of osteoporosis fractures or cognitive abilities). To date, the results of clinical research with CR confirm its safety and efficacy for menopausal symptoms and also provide valuable insights into additional uses, the mechanism of action and more.

## Conclusion

The 60 years since the launch of the first CR-based medicinal product have included preclinical and clinical research (89% of patients investigated used iCR) showing:

- Efficacy for menopausal complaints, if licensed medicinal product quality.
- Beneficial and usable already in symptomatic premenopausal patients.
- Possible supplemental benefits (increase of disease-free survival after breast cancer, adjuvant prophylaxis of osteoporosis, shrinkage of myoma, other effects).
- Safety (iCR fulfils the regulatory requirements on long-term treatment, is safe at oestrogen-sensitive tissues such as breast, uterus or tumours, did not impair tamoxifen or aromatase inhibitors, did not show hepatotoxicity in clinical studies).
- Differential effects in the periphery and influence on CNS regions responsible for thermoregulation, mood and sleep.
